# Longitudinal Multimodal Neuroimaging After Traumatic Brain Injury

**DOI:** 10.1002/hbm.70534

**Published:** 2026-04-27

**Authors:** Ana Radanovic, Keith W. Jamison, Yeona Kang, Ceren Tozlu, Sudhin A. Shah, Amy Kuceyeski

**Affiliations:** ^1^ Department of Radiology Weill Cornell Medicine New York New York USA; ^2^ Department of Computational Biology Cornell University Ithaca New York USA; ^3^ Department of Mathematics Howard University Washington DC USA

**Keywords:** flumazenil PET, longitudinal study, multimodal MRI, traumatic brain injury

## Abstract

Traumatic brain injury is a major cause of long‐term cognitive impairment, yet the mechanisms underlying recovery remain poorly understood. Neuroimaging methods such as diffusion magnetic resonance imaging (MRI), functional MRI (fMRI), and positron emission tomography (PET) provide insight into micro‐ and macro‐scale changes post‐traumatic brain injury (TBI), but the relationships between regional cellular and functional alterations remain unclear. In this exploratory study, we conducted a longitudinal, multimodal neuroimaging analysis quantifying TBI‐related pathologies in four biomarkers, namely flumazenil PET derived binding potential, diffusion MRI (dMRI)‐derived structural connectivity, and resting‐state fMRI‐derived functional connectivity and fractional amplitude of low‐frequency fluctuations in individuals with complicated mild‐to‐severe brain injury at the subacute (4–6 months post‐injury) and chronic (1‐year post‐injury) stages. The TBI sample consisted of 41 fMRI, 40 dMRI, and nine PET subjects, with 16 fMRI and dMRI and seven PET longitudinal measurements. The control sample consisted of 14 dMRI and fMRI and 19 PET subjects scanned at a single time point for comparison with TBI subjects at both time points. Most of the PET and MRI subjects are overlapping in both TBI and control groups. Brain injury related regional pathologies, and their changes over time in TBI subjects, were correlated across the four biomarkers. Our results reveal complex, dynamic changes over time. We found that flumazenil‐PET binding potential was significantly reduced in frontal and thalamic regions in brain‐injured subjects, consistent with neural loss and dysfunction, with partial recovery over time. Functional hyperconnectivity was observed in brain injured subjects initially but declined while remaining elevated compared to non‐injured controls, whereas cortical structural hypoconnectivity persisted. Importantly, we observed that brain injury‐related alterations across MRI modalities became more strongly correlated with flumazenil‐PET at the chronic stage. Regions with chronic reductions in flumazenil‐PET binding also showed weaker structural node strength and lower amplitude of low‐frequency fluctuations, a relationship that was not found at the subacute stage. This observation could suggest a progressive convergence of structural and functional disruptions with neuronal dysfunction and loss over time. Additionally, regions with declining structural node strength also exhibited decreases in functional node strength, while these same regions showed increased amplitude of low‐frequency fluctuations over time. This pattern suggests that heightened intrinsic regional activity may serve as a compensatory mechanism in regions increasingly disconnected due to progressive axonal degradation. Altogether, these findings advance our understanding of how multimodal neuroimaging captures the evolving interplay between neuronal integrity, structural connectivity, and functional dynamics after brain injury. Given the exploratory nature of this study, stemming from the modest sample size, future work in larger cohorts will be essential to validate and refine these preliminary associations as well as the inclusion of multiple measures of healthy controls. Clarifying these interrelationships could inform prognostic models and enhance knowledge of degenerative, compensatory, and recovery mechanisms in traumatic brain injury.

## Graphical abstract

We use longitudinal multi‐modal magnetic resonance imaging (MRI) and positron emission tomography (PET) imaging to examine cross‐modal relationships through the later recovery stages after brain injury. The relationships between traumatic brain injury effects on functional connectivity, fractional amplitude of low‐frequency fluctuations, structural connectivity, and GABA‐A availability proved to be dynamic and complex, with increasing convergence of MRI and PET markers over time.

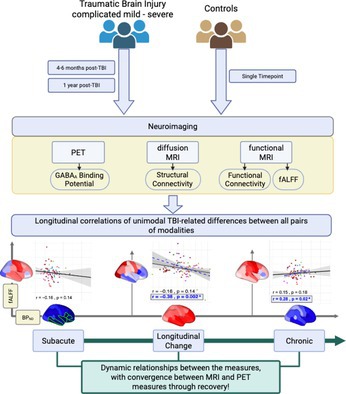

## 1 Introduction

Traumatic brain injury (TBI) is a leading cause of death and long‐term disability world‐wide. In those that survive, it is associated with multi‐scale changes in brain anatomy and physiology. Acutely, there is neuronal death or dysfunction and axonal shearing at the cellular level (Smith et al. 1997). This is followed by continued neuronal death and dysfunction, remote degeneration of structural connectivity along white matter pathways (Smith et al. 1997; Raghupathi 2004), and large scale shifts in functional co‐activation patterns that have been associated with persisting symptomology or recovery of function. To date, it is not fully understood how microscale neurobiological changes relate to macroscale connectivity, knowledge which is crucial for gaining insights into injury and recovery mechanisms. Understanding mechanisms of TBI related change is paramount to developing effective therapeutics and building more accurate prognoses.

Over 5.3 million Americans suffer from TBI‐related chronic cognitive impairments, particularly in attention, significantly impacting daily function (Stierwalt and Murray 2002; Ashman et al. 2006; Brenner 2011). Prognostic models are generally lacking in sensitivity (Silverberg et al. 2015; Rausa et al. 2020) or specificity (Le Sage et al. 2022) and there are currently few effective therapeutic interventions (Warden et al. 2006; Pink et al. 2021; Stein 2015). The failure of most TBI clinical trials has been attributed to the insufficient knowledge of the pathophysiology of specific deficits, which precludes development of targeted therapies, the inherent heterogeneity of TBI (Saatman et al. 2008) and the dearth of precise outcome markers (Silverberg et al. 2017) to track spontaneous and intervention‐driven recovery. Various neuroimaging tools, such as magnetic resonance imaging (MRI; functional and diffusion) and positron emission tomography (PET), have been used to track and identify markers of functional and structural changes following TBI. Each modality provides a unique perspective on mechanisms of recovery after TBI, but independently considered, they provide an incomplete picture.

PET is a powerful tool to investigate microscale changes post‐TBI such as neurotransmitter availability, cellular metabolism, and molecular structural features. Here we use gamma‐aminobutyric acid (GABA) receptor ligand ([11C]‐flumazenil (FMZ)) PET, a measure of GABA_A_ receptor availability that is thought to reflect neuronal dysfunction and/or loss, and has been used to study various psychiatric and neurological disorders (Ryvlin 1998; Kawai et al. 2010; Shiga et al. 2006; Kang et al. 2022; Woodrow et al. 2024). FMZ‐PET has been shown to be sensitive to losses of neuronal integrity that correlate with cognitive impairment after TBI (Shiga et al. 2006) and in the chronic stage, TBI patients with persistent cognitive impairments exhibit low FMZ uptake in frontal cortices (Kawai et al. 2010; Kang et al. 2022) and the thalamus (Kawai et al. 2010; Woodrow et al. 2024). These findings provide evidence of micro‐scale changes from subacute to chronic post‐TBI, where increases in binding might be an indication of repaired structure, or of increases in GABA_A_ activity in the remaining neurons (Frankle et al. 2012; La Fougère et al. 2011; Hughes et al. 2020; Armstrong et al. 2003; Iwakiri et al. 2009). Although these observed changes could reflect mechanisms of recovery, it remains to be understood how these microscopic changes are reflected in macroscopic structural and functional differences measured via MRI.

Diffusion MRI (dMRI) is useful in studying white matter tracts which are commonly damaged by axonal shearing and/or lesions in TBI, while functional (fMRI) is useful in assessing brain co‐activity pattern changes post‐TBI. Although various measures can be derived from these modalities, commonly structural and functional connectomes (SC and FC, respectively) are extracted. SCs are matrices that reflect the strength of white matter connections between pairs of regions in some pre‐defined atlas of gray matter structures, while FCs are matrices that reflect the co‐activation of brain activity over time between pairs of gray matter regions in an atlas. Uni‐modal connectomic analysis after TBI has revealed links between SC losses and cognitive dysfunction (Niogi et al. 2008; Dall'Acqua et al. 2016; Hulkower et al. 2013; Nikulin et al. 2011; Wang et al. 2011) and abnormal FC (either increases or decreases, depending on the study or region or network of interest) and persistent symptoms (Caeyenberghs et al. 2012; Sours et al. 2015; Woodrow et al. 2023; Mayer et al. 2011; Sharp et al. 2011; Iraji et al. 2015). Combined multimodal structural and functional connectomic analyses provide complementary information and, to date, have provided some insight into the connection between structural damage and functional outcomes (Woodrow et al. 2023; Sharp et al. 2011; Dall'Acqua et al. 2017; Kuceyeski et al. 2019; Parsons et al. 2023; Xiao et al. 2015). However, the heterogeneic and dynamic nature of TBI, as well as the effect of varied image acquisition, processing strategies, and inherent noise properties of f/dMRI, has led to conflicting results when mapping connectome changes to outcomes after TBI.

While resting state FC reveals co‐activation changes between regions, fractional amplitude of low‐frequency fluctuations (fALFF) provides complementary functional information regarding regional activity as it is a marker of the level of spontaneous neural activity (Zou et al. 2008). fALFF has been found to be increased in TBI patients (Vedaei et al. 2021; Qi et al. 2010; Han et al. 2011; Zhan et al. 2016), possibly reflecting neural dysfunction or excitotoxicity after TBI. Studies have linked increases in fALFF to increases in binding of fluorodeoxyglucose F‐18 (FDG) and FMZ PET in controls (Aiello et al. 2015; Rajkumar et al. 2021; Deng et al. 2022), though the relationship is not well defined. fALFF's relationship to connectomic measures has generally not been explored, especially in TBI, though fALFF and FC have shown positive correlations in order conditions such as autism (Itahashi et al. 2015). None of these handful of studies examine intermodal relationships between fALFF and structural (SC), functional (FC), or FMZ‐PET measures in TBI, let alone across recovery.

Intermodal relationships between MRI and PET markers (other than FMZ), such as FDG‐PET (glucose metabolism), have demonstrated utility in identifying differential recovery trajectories in mild and moderate TBI subjects (Ito et al. 2016), elucidating chronic cognitive impairments in blast TBI (Stout et al. 2013), and predicting recovery in severe cases of TBI (Annen et al. 2016). Although multi‐modal MRI and FMZ‐PET analyses have provided insights into other conditions such as multiple sclerosis (Freeman et al. 2015), their relevance to TBI recovery remains largely unexplored. In healthy non‐injured adults, tri‐modal studies (FMZ‐PET, MRI, EEG) have linked increases in FMZ‐PET binding to increases in resting fMRI measures, including fALFF, across networks like the executive control network (Rajkumar et al. 2021). Also, the utilization of multi‐modal MRI and PET metrics has shown promise in distinguishing chronic TBI patients from healthy controls (Vedaei et al. 2024). These findings suggest joint FMZ‐PET and MRI studies in TBI could reveal microscale (PET) and macroscale (MRI) mechanisms underlying recovery. Advancing knowledge of multi‐scale mechanisms of TBI recovery trajectories requires the integration of microscale PET data with macroscale MRI features.

Here, we set out to explore the relationship between diffusion and functional MRI metrics and FMZ‐PET metrics longitudinally across the post‐TBI recovery period to garner a more comprehensive understanding of multi‐scale recovery mechanisms. We collected longitudinal PET and MRI in individuals with TBI at “subacute” (4–6 months post‐TBI) and “chronic” (1 year post‐TBI) stages; imaging from non‐injured controls was also collected. From each scan, we extracted multi‐scale, regional biomarkers of brain anatomy and physiology at two levels: cellular, that is, FMZ‐PET binding potential (BP_*ND*_), and macroscale, that is, diffusion MRI structural connectivity (SC), resting‐state fMRI functional connectivity (FC) and fractional amplitude of low‐frequency fluctuations (fALFF). We quantified TBI‐related pathologies (compared to the non‐injured controls) in the four biomarkers at both time points, as well as the change in these TBI‐related pathologies over time. TBI‐related regional pathologies, and their changes over time, were correlated across modalities. This paper is the first to explore and compare cellular and macro‐scale metrics of TBI‐related pathologies to one another, and further, to investigate the evolution of these across‐modality relationships over time.

## 2 Materials and Methods

### 2.1 Study Design

Multi‐modal flumazenil PET (FMZ‐PET), diffusion (dMRI), and resting‐state functional MRI (fMRI) were collected in individuals with TBI and non‐injured healthy controls (HC). TBI subjects were imaged at 4–6 months after injury (mean = 150.5 days for MRI and 140 days for PET) and again 12 months after injury (average time between scanning sessions was 253 days for MRI and 297 days for PET). These timepoints are referred to as “subacute” and “chronic” for clarity in presentation; however, we acknowledge that both may fall within the chronic phase, depending on the recovery timeline definition. HCs were imaged once for comparison to the TBI group at both time points, whereas longitudinal analyses were restricted to the TBI group. FMZ‐PET data was collected from seven TBI patients (four female, aged 33–58, mean 48 years) and 19 HCs (seven female, aged 22–65, mean 44 years). dMRI and fMRI data were collected from 16 TBI patients (four female, aged 19–73, mean 48 years) and 14 HCs (five female, aged 23–86, mean 56 years); the seven TBI PET subjects are a subset of the 16 TBI MRI subjects, and 9 HC PET subjects are a subset of the 14 MRI. Further demographic breakdown for TBI subject injury measures, sample size, sex, and age for each group and time point can be found in Tables S1 and S2.

### 2.2 Participants and Recruitment

TBI participants were recruited through inpatient rehabilitation units and trauma departments at large, urban academic medical centers. Control individuals were recruited through local advertisements. All study activities were approved by Weill Cornell Medicine's Institutional Review Board.

All participants were required to meet the following criteria: (i) 18 years of age or above; (ii) English‐speaking; (iii) capable of providing informed consent or a proxy/authorized agent available to provide informed consent; (iv) physically healthy and able to safely undergo PET and/or MR imaging; (v) not currently taking any psychoactive or benzodiazepine drugs; (vi) not currently taking any medication for attention‐deficit/hyperactivity disorder; (vii) no history of schizophrenia, drug, or alcohol abuse; (viii) no history of epilepsy, stroke, dementia, or serious medical illness by self‐report; and (iv) not pregnant (for female participants). Participants in the TBI group were recruited if they sustained a complicated mild (Glasgow Coma Scale 48 score of 13–15 with evidence of intracranial lesion as verified on acute neuroimaging) or moderate–severe TBI (Glasgow Coma Scale score *≤* 12) within the last 6 months.

### 2.3 PET Acquisition and Processing

We measured brain GABA_A_ receptor binding using PET imaging with the radioligand ethyl 8‐fluoro‐5,6‐dihydro‐5‐[11C] methyl‐6‐oxo‐4H‐imidazo [1,5‐a] [1, 4] benzodiazepine‐3carboxylate, or [^11^C]‐FMZ. A dynamic PET scan was acquired for 60 min beginning at injection, in 3D list mode with the same whole‐body PET/CT scanner (mCT, Simens/CTI, Knoxville, TN) with a spatial resolution of ∼4 mm measured as the reconstructed full‐width at half maximum of a point source in air. The acquired scans were corrected for photon absorption and scatter, using an in‐line CT scanner (120 kV, 30 mA, pitch = 1.5). PET data were reconstructed into 22 frames (4 × 15 s, 4 × 30 s, 3 × 60 s, 2 × 120 s, 8 × 300 s, 1 × 600 s) using the vendor‐provided iterative + time‐of‐flight list‐mode algorithm, with a 400 × 400 matrix, voxel size 1.082 × 1.082 × 2.025 mm^3^, and zoom = 2.0. Summed PET images were coregistered to each participant's structural MRI using rigid registration and mutual information.

Time‐activity curves were extracted from each region of interest. Binding potential (BP_*ND*_) to GABA_A_ receptors, a proxy measure for neuronal integrity, was extracted from the FMZ‐PET images using PMOD software that implements the Logan graphical model with pons acting as the reference region. See Kang et al. (2022) for more details on the PET acquisition and processing used in this study, though Kang et al. (2022) used a simplified reference tissue model to estimate BP_*ND*_, rather than Logan graphical analysis.

### 2.4 MRI Acquisition and Preprocessing

A 3T Siemens Prisma scanner with a 32‐channel head coil was used to collect anatomical, functional, and diffusion‐weighted MRI images. The protocol was adapted from the Human Connectome Project Lifespan study (Harms et al. 2018). Anatomical images included 3D T1‐weighted sagittal MPRAGE and T2‐weighted SPACE (0.8 mm isotropic voxels). Resting‐state functional MRI was acquired with 2 mm isotropic voxels, 72 axial oblique slices, 420 volumes (TR/TE = 800/37 ms, multi‐band factor 8), with a total acquisition time of 11 min, 12 s, divided between two scans with opposite phase encoding directions (AP = anterior‐to‐posterior, and PA = posterior‐to‐anterior). A matching pair of spin echo field maps with opposing phase encoding direction was collected for each resting state scan. Multi‐shell diffusion‐weighted MRI was acquired with 1.5 mm isotropic voxels, 92 axial oblique slices (TR/TE = 3230/39.2 ms, multi‐band factor 4), *b* = 1500/3000, 92 directions per shell, acquired in both AP and PA phase‐encoding directions for a total acquisition time of 22 min 36 s.

MRI data was preprocessed using the Human Connectome Project Minimal Preprocessing Pipeline (Glasser et al. 2013). Anatomical images were inhomogeneity‐corrected, anatomically segmented using FreeSurfer 6.0, and nonlinearly registered to the MNI152 template (6th generation). Functional MRI was motion‐corrected, EPI distortion corrected using FSL's “topup” (Smith et al. 2004), linearly coregistered to the anatomical image, and resampled to MNI152 space. Diffusion MRI was jointly corrected for motion, EPI distortion, and eddy current distortion using FSL's “eddy” tool (Andersson and Sotiropoulos 2016), before being linearly coregistered to the anatomical image. Regional analyses used FreeSurfer‐generated, subject‐specific 86‐region atlases (68 cortical gyri from Desikan‐Killiany and 18 subcortical gray matter regions) (Desikan et al. 2006; Fischl et al. 2002).

### 2.5 Resting‐State fMRI Processing

Preprocessed functional MRI time series were denoised using custom scripts to identify outlier time points (motion derivative threshold 0.9 mm, global signal threshold 5*σ*), regress out motion and tissue‐specific nuisance time series (24 motion time series (Power et al. 2014) and 10 eigenvectors derived from eroded white matter and CSF masks (Behzadi et al. 2007)), and temporally filter the final result (high‐pass filter cut‐off 0.008 Hz, using DCT projection). Outlier timepoints were excluded from nuisance regression and temporal filtering. Regional time series for each of the 86 gray matter areas were obtained from the denoised time series data. Pearson correlation between regional time series (excluding outlier timepoints) resulted in an 86 × 86 resting‐state functional connectivity (FC) matrix for each AP and PA scan, which were then averaged together. Functional connectivity node strength for each region was computed as the sum of the positive elements in each row of the FC matrix (excluding negatives and diagonal).

fALFF is a metric that measures the relative contribution of the power of low‐frequency blood‐oxygen‐level‐dependent (BOLD) signal fluctuations which are assumed to reflect the magnitude of neural activity (Zou et al. 2008). Spatial smoothing is first performed for every subject. Fast Fourier transform (FFT) was used to convert the signal to the frequency domain and the square root of the power spectrum was averaged across the 0.01–0.09 Hz domain. fALFF is the ratio of power in low‐frequency band (0.0008–0.09 Hz) to the power of the entire frequency range (0.008–0.625 Hz). fALFF was calculated at a voxel‐wise level and then averaged to obtain regional fALFF.

A supplementary analysis was conducted to account for the increase in low frequency oscillation strength with increased scanner time, whereby rapidtide (Frederick et al. 2026) (v3.1.1) was used to preprocess the fMRI data prior to fALFF calculation (see Figure S5). Rapidtide identifies and removes time‐varying physiological blood signals from fMRI data by performing time‐lagged cross‐correlation analysis between voxelwise BOLD timeseries and a moving systemic low frequency oscillation (sLFO) regressor derived from the data itself, without requiring external physiological recordings. The denoising procedure used an sLFO (0.01–0.15 Hz) global time series derived from gray matter voxels, and was applied within a brain mask, with motion parameters included as additional confound regressors. A temporal exclusion mask was applied to exclude specified time points from the sLFO regressor estimation, ensuring that periods of excessive motion or other artifacts did not contribute to the regressor derivation. The voxel‐specific delayed sLFO regressor was then regressed out of the original, unmodified fMRI data, such that the only filtering applied to the final output was removal of the moving sLFO signal. fALFF was then calculated via the procedure mentioned above.

### 2.6 Diffusion MRI Processing

Preprocessed diffusion MRI were further processed using MRtrix3 (Tournier et al. 2019), including bias correction, constrained spherical deconvolution (multi‐shell, multi‐tissue FOD estimation, lmax = 8 (Jeurissen et al. 2014)), and whole‐brain probabilistic tractography (iFOD2 (Tournier et al. 2010)) using uniform white matter seeding (20 seeds per white matter voxel, resulting in approximately 5 million total streamlines per subject). Structural connectivity (SC) matrices were constructed by counting the number of streamlines that ended in each pair of gray matter regions, normalized by the total volume of each region pair. To mitigate the problem of false positive connections, region‐pairs with zero streamlines in at least 50% of the uninjured control subjects were replaced with zeros throughout the dataset. Structural connectivity node strength for each region was computed as the sum of each row in the SC matrix (excluding the diagonal).

### 2.7 Statistics

Subacute (denoted as ses 1) and chronic (denoted as ses 2) analyses of each imaging metric were conducted via one‐way analysis of covariance (ANCOVA) where *t*‐statistics of coefficients' group effects were examined as the primary effect of interest, with age and sex as covariates. All available healthy controls for a given modality were included in these ANCOVAs, which varied according to modality (see Figure 2, *n* = 7 TBI subjects had both MRI and PET). We included all available controls to increase power in determining TBI‐related effects for a single modality, with the assumption that healthy controls are drawn from the same distribution within the population.

A second set of ANCOVAs was performed using the subset of TBI subjects that had both MRI and PET data (*n* = 7) in order to compare MRI and PET measures. Longitudinal change within TBI subjects was analyzed using linear mixed effects models for only those with two time points (PET: *n* = 7; MRI: *n* = 16 TBI), with sex and age as covariates. Because healthy controls were imaged at a single time point, longitudinal analyses were conducted exclusively in the TBI group.

**FIGURE 1:**
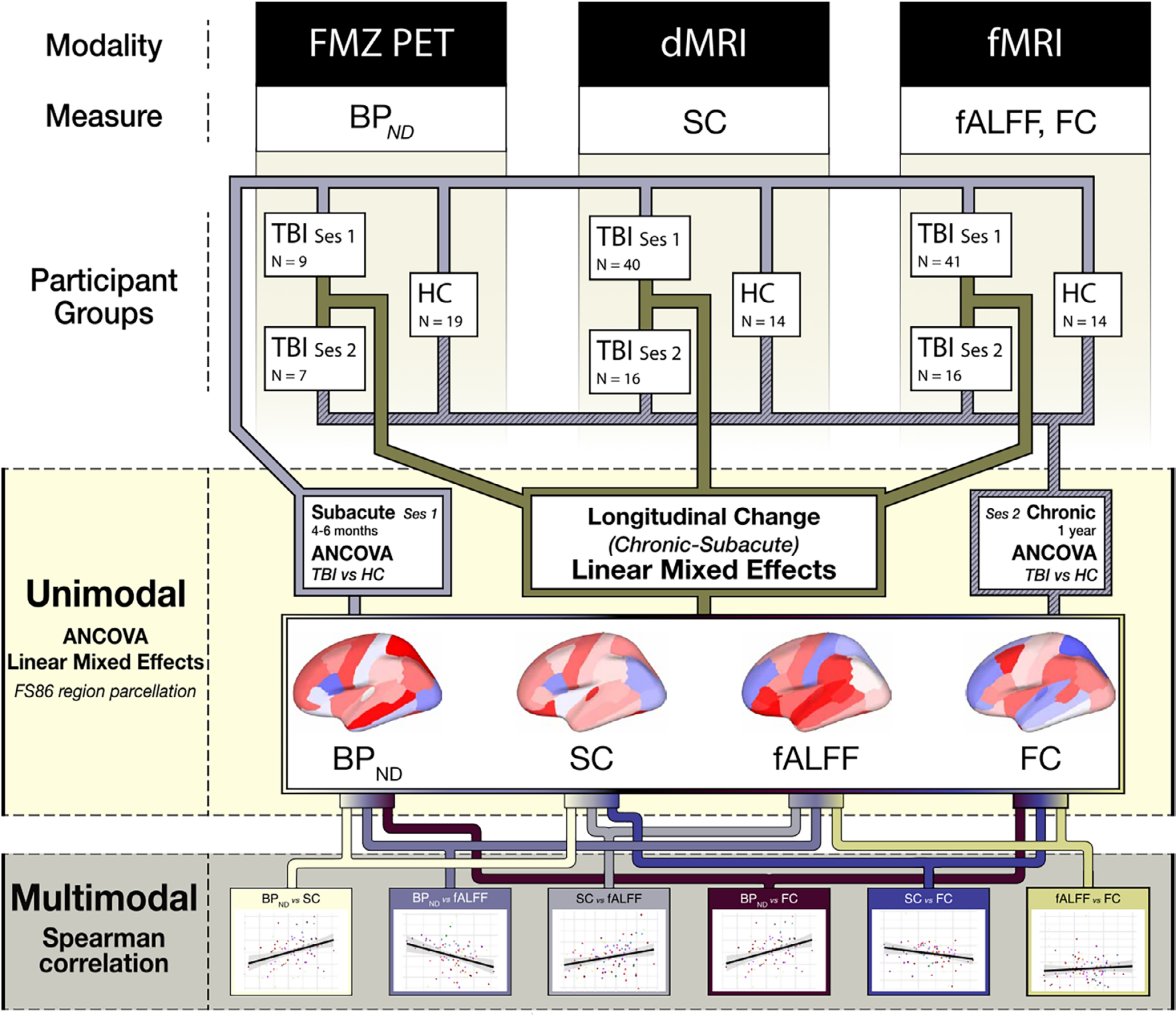
Analysis workflow for longitudinal multi‐modal analysis. Between the three modalities (PET, dMRI, and fMRI) four biomarkers per brain region were obtained—namely, BP_*ND*_ from PET, SC node strength from dMRI, and fALFF and FC node strength from fMRI. These were extracted from all participant groups. TBI subjects have two sessions of recording 6–8 months apart with a single session from healthy controls. The number of subjects in each group and time point are denoted by *N*. ANCOVA was applied to uncover the effect of TBI on the various markers in the subacute (first) and chronic (second) session as compared to healthy, non‐injured controls. A linear mixed effects model between the two sessions was used to investigate changes over time in the TBI subjects. The *t*‐statistics for the group coefficients (TBI vs. controls) in ANCOVA and the coefficients from the linear mixed effects models from each of the four biomarkers are then Spearman correlated in a multimodal analysis.

Multimodal analyses were performed as follows: For each modality, group *t*‐statistics from ANCOVA (TBI vs. control) and coefficients for the session effect from linear mixed‐effects models were correlated using Spearman rank correlations, with *p*‐values calculated using 1000 permutations. All statistic and *p*‐values reported are uncorrected for multiple comparisons, as significant results did not survive multiple comparison correction. The analysis pipeline and methodology summary can be seen in Figure 1. Data were analyzed and visualized using custom scripts in using R (http://www.r‐project.org) version 4.4.3 and Python.

## 3 Results

**FIGURE 2:**
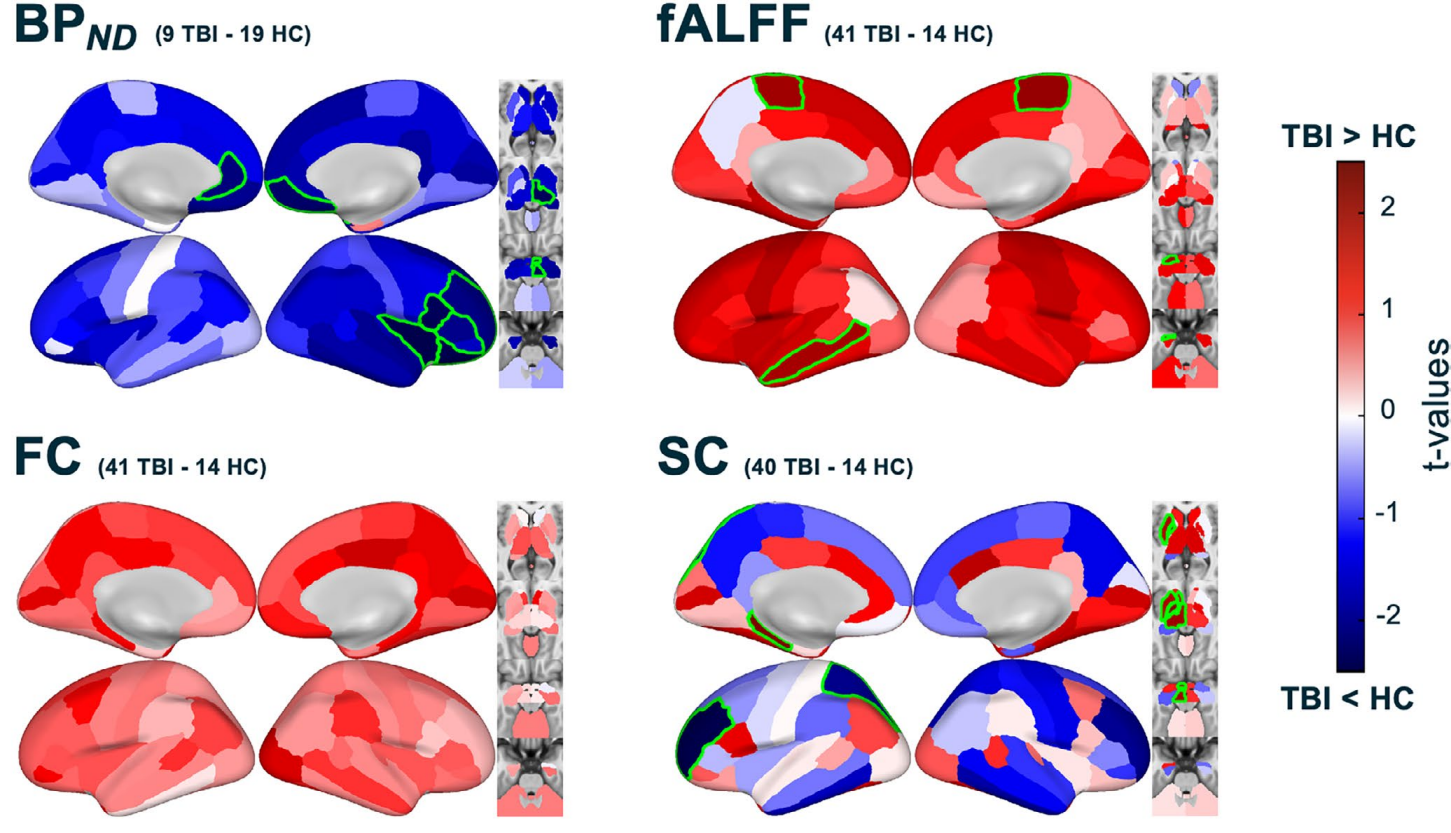
Group differences in [^11^C] FMZ tracer binding potential (BP_*ND*_), functional activity level (fALFF), functional connectivity (FC) node strength, and structural connectivity (SC) node strength in individuals with TBI compared to non‐injured controls at the subacute timepoint. Subacute timepoint *t*‐values of group coefficients from ANCOVA with age and sex as covariates. *N* varies by modality, as indicated in each subplot. Significant (uncorrected, *p* < 0.05) values are highlighted in green.

Group (TBI vs. HC) coefficients' *t*‐statistics for each of the four biomarkers were plotted for each of the 86 regions for the subacute time point, see Figure 2. As reported before (Kang et al. 2022), [^11^C] FMZ—BP_*ND*_ was generally lower in TBI subjects compared to controls, particularly in frontal and temporal regions as well as subcortical structures (*p <* 0.05). fALFF and FC exhibited the opposite pattern, in which TBI subjects showed generally higher values than healthy controls at the subacute timepoint. fALFF was significantly higher in sensorimotor and temporal regions (*p <* 0.05) while FC was most elevated in occipital and posterior cingulate, though not reaching significance. Results for SC are mixed, with the cortex having generally lower structural connectivity (frontal, para‐hippocampal, and parietal regions) reaching uncorrected significance (*p <* 0.05). A few regions in the left subcortex, including thalamic areas, reflect higher SC in TBI than HCs (*p <* 0.05).

### 3.1 Longitudinal Unimodal Analysis

**FIGURE 3:**
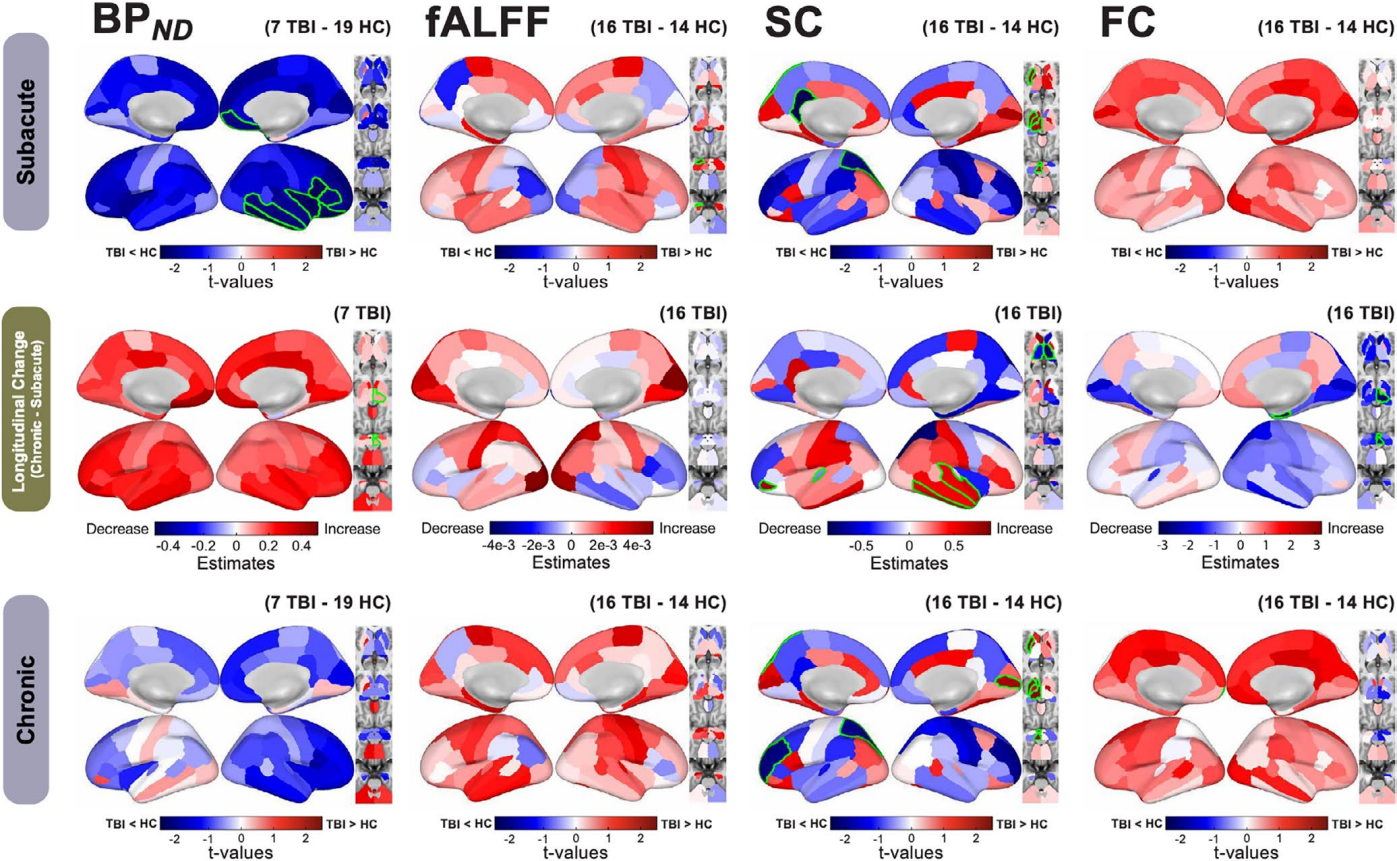
Longitudinal analysis of group differences (TBI vs. controls) in regional FMZ tracer binding potential BP_*ND*_, functional activity strength (fALFF), structural connectivity node strength (SC), and functional connectivity node strength (FC). At baseline timepoint (Subacute—first row), longitudinal change (second row), and follow‐up time‐point (Chronic—third row). Subacute and chronic timepoints are visualized via *t*‐values of group coefficients from ANCOVA and longitudinal changes are visualized via coefficients of the session effect of linear mixed effects model analysis. *N* varies by modality, as indicated in each subplot. Regions with significant (*p <* 0.05) values are outlined in green, uncorrected.

For the longitudinal analysis, an ANCOVA was performed on a smaller subset of TBI subjects who had data collected at both time points (*n* = 7 PET, *n* = 16 MRI), see first and last rows of Figure 3. As a result, the acute results in the first row of Figure 3 differ slightly from the acute results in Figure 2, as the subject pool for MRI drops from 41 subjects to 16, and from 9 to 7 for PET. However, the subacute results in the first row generally match the full dataset subacute results in Figure 2, apart from some regions having lower fALFF in TBI vs. controls.

The longitudinal change was analyzed looking only at the TBI subject's change over time via a linear mixed effects model, see the middle row of Figure 3. As reported previously (Kang et al. 2022), subjects with TBI had increases in cortical BP_*ND*_ from subacute to follow‐up; however, these increases were not large enough, generally, to return to the levels of non‐injured controls, particularly in frontal regions. In the subcortex, although not significant, TBI subjects exhibit higher BP_*ND*_ compared to controls in the cerebellum and left thalamus at the chronic stage. fALFF results did not reach significance; however, they were initially higher in frontal regions and lower in parietal regions, but over time, fALFF in frontal regions (particularly right hemisphere) and most subcortical regions decrease; there are concomitant increases in parietal regions.

Although low frequency oscillation strength has been shown to artifactually increase with scanning time (Korponay et al. 2024), fALFF findings were similar when data was corrected using rapidtide (Korponay et al. 2024) (see Figure S5). In addition, linear mixed effects models were run including the number of days between scans as a random slope, and the unimodal and multimodal (discussed below) results were similar (see Figure S6).

SC is generally lower in the subacute timepoint compared to controls, with more increases (trends toward significant changes in right temporal areas) than decreases over time, and generally still persistently lower SC at chronic stages, though less severe than in subacute stages. Patterns of TBI group differences in SC were very similar from subacute to chronic stages (see first and last row of Figure 3). Of note, in both the subacute and chronic timepoints, SC was significantly higher in the visual cortex, significantly so at chronic (*p <* 0.05). Thalamic regions that had initially higher SC at the subacute timepoint showed significant decreases in SC over time, while the putamen and other subcortical regions remained significantly higher compared to controls from subacute to chronic. Although not significant, FC was higher in TBI subjects compared to controls at the subacute timepoint, with reductions over time. Despite reductions in cortex FC over time, FC was still persistently larger than controls at the chronic timepoint. However, for some subcortical regions, the reductions over time were enough to result in lower FC compared to controls at chronic.

### 3.2 Multimodal Longitudinal Analysis of TBI‐Related Effects

**FIGURE 4:**
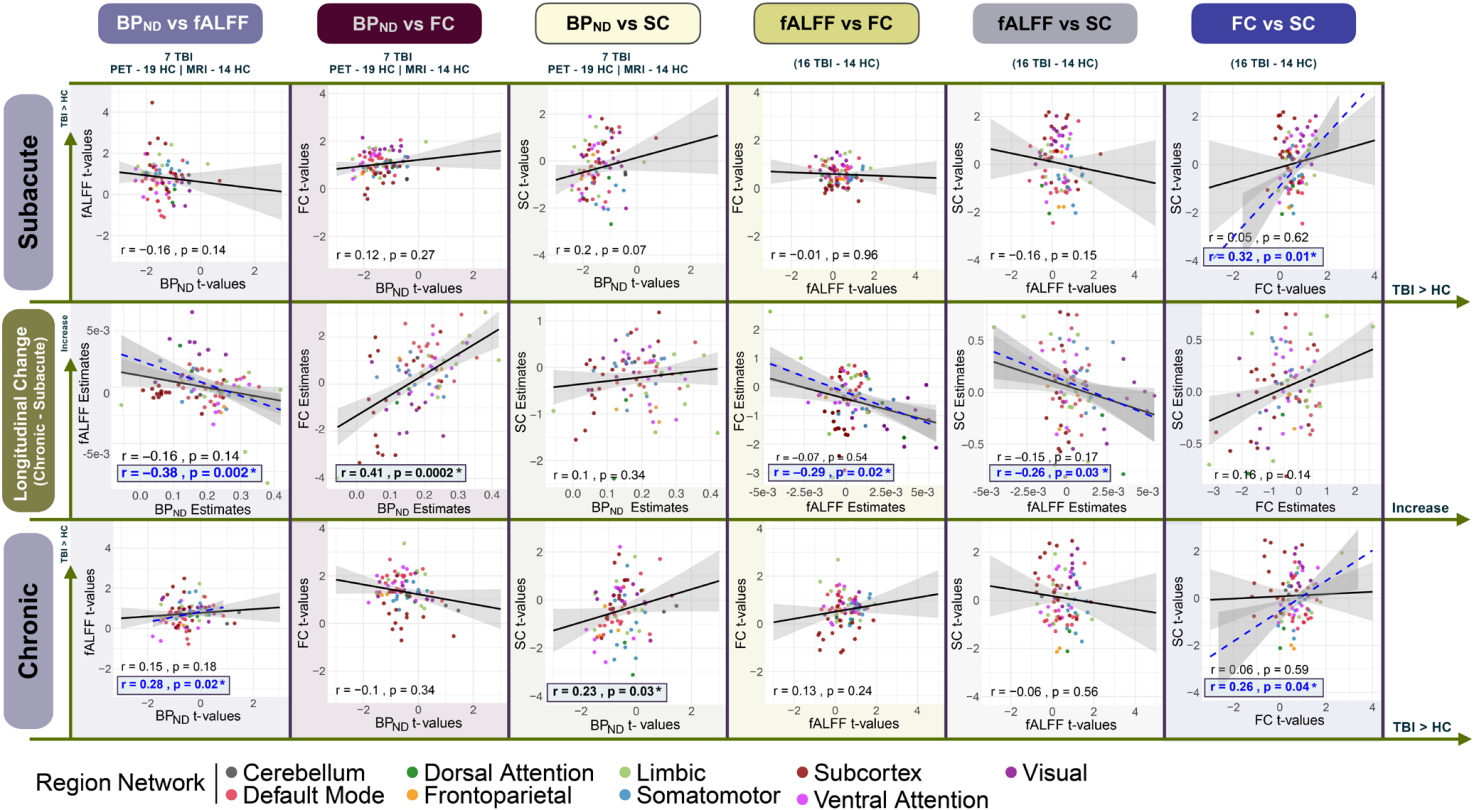
Inter‐modality comparison of TBI‐related changes in imaging metrics. Spearman correlations of the *t*‐values from ANCOVA group coefficients (TBI vs. control) between FMZ tracer BP_*ND*_, fALFF, FC, and SC at baseline (Subacute—first row) and follow‐up (Chronic—third row). Session effect coefficients indicating change vs. change for each pair of modalities are in the middle row. Correlations are calculated for all brain regions or, where indicated, cortical regions only (in blue). Cortex‐only correlations (dashed blue line) are only shown for comparisons where it differs substantially from the whole‐brain correlation (solid black line). Correlation strength and *p*‐value indicated in bottom corner, asterisk and bold indicate significance (uncorrected, *p <* 0.05). *N* varies by modality, indicated under titles of paired modality columns. Each region is colored by the functional network to which it belongs (see legend).

Finally, we examined correlations of TBI‐related differences in the four imaging biomarkers. As with the previous longitudinal analysis, only TBI subjects who had both sessions available were included (*n* = 7 PET, *n* = 16 MRI). Group ANCOVA coefficients' (TBI vs. HC) *t*‐values from the multiple biomarkers at each time point were correlated via Spearman rank correlations; session effect coefficients from the TBI only linear mixed effects models reflecting the changes in individual biomarkers were also Spearman rank correlated to change in the other biomarker, see Figure 4. These values were correlated to examine how the group and session effects are correlated between the modalities, with sex and age accounted for.

In the first column, changes in BP_*ND*_ and fALFF are negatively correlated (*r* = −0.38, *p* = 0.002) while the two biomarkers are positively correlated in the chronic time point in the cortex (*r* = 0.28, *p* = 0.02), such that regions with larger TBI‐related decreases in binding potential also had fALFF values that were not as elevated in TBI compared to other regions. Trending but not significant at the subacute time point, this relationship is opposite to what is seen in the subacute time point, where regions with more TBI‐related decreases in BP_*ND*_ also had more elevated fALFF.

In the second column, change in BP_*ND*_ and change in FC in cortical regions are positively correlated (*r* = 0.41, *p* = 0.0002), indicating regions with larger increases in FMZ‐binding also had larger increases in FC. In the third column, BP_*ND*_ and SC are positively correlated at chronic timepoint (*r* = 0.23, *p* = 0.03) and trended toward significance at the subacute timepoint, such that regions with larger TBI‐related decreases in binding also had more SC damage. In the fourth column, fALFF and FC are not correlated at subacute or chronic timepoints; however, the longitudinal changes in fALFF and FC were negatively correlated (*r* = −0.29, *p* = 0.02) in cortical regions such that regions with more increases in fALFF over time also had larger decreases in FC. Although not significantly negatively correlated at the subacute and chronic timepoints, the longitudinal change in fALFF and SC were negatively correlated in the cortex (*r* = −0.26, *p* = 0.03) such that regions with decreasing SC had increasing fALFF. In the final column, FC and SC are positively correlated in cortical regions at both subacute (*r* = 0.32, *p* = 0.01) and chronic timepoints (*r* = 0.26, *p* = 0.04), with longitudinal change within regions trending toward a positive correlation meaning regions with larger TBI‐related decreases in SC also had less TBI‐related elevations in FC.

### 3.3 Robustness of Multimodal Analyses and Cognitive Outcome Correlations

We provide additional details on the main results in the Supporting Information, including robustness analyses and global correlations between imaging metrics, cognitive scores, and outcome measures. First, ANCOVA *η* ^2^ results for group effects of *t*‐values plotted in Figures 2 and 3 are shown in Figure S3. Next, we performed a multi‐modal correlation analysis on a larger cohort, including all available subjects at the subacute timepoint, to increase statistical power. The *t*‐values from Figure 2 were correlated across modalities (see Figure S1). Consistent with findings from the smaller cohort (Figure 4), TBI‐related differences in FC and SC node strength were positively correlated in the cortex. Trends toward positive correlations between TBI‐related BP_*ND*_ and SC node strength were observed in both cohorts, though neither reached significance. These results suggest consistency in the observed relationships across different sample sizes. Additionally, we examined global average imaging metrics in relation to cognitive and standard TBI outcome measures. Overall, no significant associations were found, except for a few uncorrected relationships where increased fALFF and BP_*ND*_ were linked to improvements in GOSE scores over time. However, the relationships between individual global metrics and various outcomes at subacute and chronic timepoints were inconsistent and did not reflect a clear pattern across different outcome measures (see Figure S4).

## 4 Discussion

Here we use multi‐modal PET and MRI imaging in an exploratory analysis to understand multi‐scale structural and functional changes during recovery from TBI. While our unimodal findings align with prior research, we find that the across‐modality relationships of TBI‐related differences allow deeper insight into the complex and dynamic recovery process. Overall, the across modality (PET‐MRI) relationships were stronger in the chronic stage. We found that regions with chronic, persistent decreases in BP_*ND*_ due to TBI also had weaker SC node strength and less elevated neuronal activity measures, a relationship that wasn't found at the subacute time point, suggesting a convergence of structural and functional disruptions driven by neuronal loss and/or dysfunction over time. We also found that regions with TBI‐related decreases in SC node strength also had TBI‐related decreases in FC node strength, and furthermore, regions with decreasing SC and FC node strength over time also had increases in a measure of neural activity (fALFF) over time. The latter potentially indicating neural hyperactivity, excitability, changes in neurovascular BOLD response from injury (Wu et al. 2019), or a compensatory mechanism in regions that are increasingly disconnected from the rest of the brain.

### 4.1 Longitudinal Unimodal Changes During Recovery

As shown before by our group and others (Kang et al. 2022; Woodrow et al. 2024), we found lower BP_*ND*_ in TBI subjects compared to controls, with an increase over time to levels that were still generally lower than controls, particularly in frontal regions and the thalamus. It has also been found that lower binding in thalamic regions is associated with worse outcome after TBI (Woodrow et al. 2024). Importantly, some regions that had significantly lower binding such as the anterior cingulate cortex (ACC) are heavily implicated in attention and emotional processing, which are common afflictions post‐TBI (Ashman et al. 2006; Kim et al. 2023; Shah et al. 2017; Bornhofen and Mcdonald 2008; Halterman et al. 2006).

Functional connectivity analyses in resting state fMRI after TBI have been conflicting (Morelli et al. 2021; Shumskaya et al. 2012; Stevens et al. 2012). Typically analyzed via seed or ICA based methods, studies report resting state hyperconnectivity (Sours et al. 2015; Dall'Acqua et al. 2017; Caeyenberghs et al. 2017) in the very acute stages (within 2 weeks of injury) between the thalamus and the pre‐frontal cortex (Sours et al. 2015; Woodrow et al. 2023), and the DMN and the frontal cortex (Mayer et al. 2011). Other research suggests that hypoconnectivity within the DMN and hyperconnectivity with the DMN and other regions at rest in acute and chronic stages, that can predict persistent cognitive symptoms (Sharp et al. 2011; Iraji et al. 2015). At 6 months after injury, higher FC is associated with lower cognitive impairment (Sharp et al. 2011). Generally, however, our results are not entirely consistent with previous findings, as we did not find significant FC differences, only trends of higher FC was found after TBI at both time points—at least in the cortex (see Figures 2 and 3). One thought is that the brain attempts to compensate for structural and functional disruptions by recruiting resources from other areas, that is, functional compensation (Iraji et al. 2015; Dall'Acqua et al. 2017; Vedaei et al. 2021; Caeyenberghs et al. 2017; Guo et al. 2019).

Despite only trends of generally higher FC in the cortex at both time points, subcortical structures showed significant decreases over time, with some regions having lower FC compared to controls at the chronic time point (though not significant). This could potentially reflect increased vulnerability of the connections between subcortical and cortical regions (Woodrow et al. 2024) that worsen over time and result in their decoupling. However, aside from heterogeneity in TBI severity and localization, different choices in fMRI preprocessing (i.e., global signal regression; GSR) and calculation of FC (seed‐based, atlas‐based, correlation type, etc.) complicates cross‐study comparisons.

Increasing fALFF over time has been correlated with improved symptom scores from 0 to 3 months after TBI (Madhavan et al. 2019), complementary to a finding of lower fALFF in the thalamus, frontal, and parietal regions at < 25 days after injury in subjects with post‐concussive symptoms (Zhou et al. 2014). Our analysis in the larger subject pool (Figure 2) showed subjects had generally higher fALFF across cortical regions, except for occipital regions, some subcortical areas, and cerebellum at the subacute stage, though this did not reach significance. Individually, higher fALFF and FC values are thought to possibly reflect compensatory mechanisms for structural damage (Iraji et al. 2015; Dall'Acqua et al. 2017; Vedaei et al. 2021; Caeyenberghs et al. 2017; Guo et al. 2019; Stephenson et al. 2020), or changes in neurovascular coupling which have been found in severe brain injury (Wu et al. 2019), and reduction in these values over time may signal an exhaustion of these compensatory mechanisms, or a relief due to recovery. Large, comprehensive longitudinal studies with (1 year+) multiple measured timepoints may be able to better capture the dynamics of these mechanisms.

SC node strength was generally lower in TBI compared to controls at both subacute and chronic timepoints, see Figures 2 and 3, in line with the idea that injury induces not only white matter tract damage but also progression of continued degeneration along axonal tracts (Povlishock and Katz 2005). SC analysis has often found hypoconnectivity due to damage (Dall'Acqua et al. 2016), particularly in temporal and frontal white matter tracts, that has been associated with cognitive dysfunction (Moreira Da Silva et al. 2020). SC hyperconnectivity has also been identified in “central hub areas” in very acute timepoints, which could either reflect the effects of acute edema (Hutchinson et al. 2018) or potentially effects of functional reorganization with information processing to higher hubs, akin to rich club networks (Dall'Acqua et al. 2017). We found that some regions in the visual cortex and left putamen exhibited higher SC over time in TBI compared to controls, with regions in the right (and to a lesser extent left) temporal lobe having increases in SC over time, which could be reflective of either repair to white matter tracts or changes in response to the widespread functional upregulation. The thalamus showed trends toward initially increased SC with significant decreases in SC over time, in line with findings that the thalamus, although not necessarily impacted directly by the initial insult, will show progressive neuronal and structural loss through TBI recovery (Woodrow et al. 2024). SC differences are also difficult to tease apart as well because of the heterogeneity in TBI etiology and spatial patterns of axonal injury across subjects.

### 4.2 Multi‐Scale PET and MRI Relationships

Very few studies have related FMZ‐PET to (f)MRI metrics. One prior research finding in controls using simultaneous PET‐MR‐EEG imaging found network level correlations across individuals between fALFF and BP_*ND*_ (Rajkumar et al. 2021), wherein higher fALFF was related to higher BP_*ND*_. Other research found a positive correlation between BP_*ND*_ and increases in FC in the visual cortex between changes in eyes open and eyes closed conditions at rest (Qin et al. 2012). Here, we identified a positive correlation between TBI‐related pathology in fALFF and BP_*ND*_ at the chronic timepoint, opposite to the trending relationship at the subacute timepoint. This somewhat paradoxical finding could reveal neural dysfunction that results in hyperactivity or impaired neurovascular coupling at the subacute point, which, alongside chronic neurodegeneration (Smith et al. 1997), exhausts at the chronic stage more dramatically for those neurons with dysfunction or death (decreased BP_*ND*_) resulting in lower levels of neuronal activity reflected by lower fALFF. In other work, a related measure called ALFF was shown to be positively correlated with FMZ‐PET and glucose metabolism (rmGLU)‐PET in healthy controls and some subjects with temporal lobe epilepsy (TLE), but not in all subjects. As ALFF was found to be more highly correlated with rmGLU than FMZ‐PET BP_*ND*_, decoupling of ALFF and FMZ‐PET may be due to impaired neurovascular coupling (Nugent et al. 2015) which could be a factor more salient subacutely in our TBI population (Jullienne et al. 2016).

FMZ‐PET binding has been shown to correlate with MRI‐based atrophy in epilepsy (Juhász et al. 1999) and with T2‐weighted lesion load in multiple sclerosis (Freeman et al. 2015), and to possibly capture neuronal loss not visible on anatomical MRI (Shiga et al. 2006). However, the relationship between FMZ‐PET binding and dMRI‐based SC has been explored in only a handful of studies. One such prior study found that individuals with lower thalamic FMZ‐PET binding had more lesions in the white matter connecting to the thalamus (i.e., weaker SC) (Woodrow et al. 2024). We found an alignment of this previous work at the chronic timepoint, BP_*ND*_ and SC were positively correlated, that is, regions with more TBI‐related decreases in FMZ‐BP_*ND*_ also had more decreases in SC node strength (see Figure 4). This relationship only reached significance at the chronic time point and was only trending at the subacute time point, which could be due to continued neuronal degeneration after TBI that resulted in a strengthening of the relationship between the two metrics. TBI effects on MRI and PET measures are generally more correlated at the chronic timepoint. Our conjecture is that there is perhaps TBI‐related neuronal loss and changes in neuronal function occurring over time, which is partly reflected in persistently lower BP_*ND*_, longitudinally decreases, but still leads to higher‐than‐normal fALFF and weakening SC node strength.

### 4.3 MRI‐Based Structure–Function Relationships in TBI

FMRI provides information on brain activity (e.g., fALFF) and co‐activity patterns (FC), while diffusion MRI provides estimates of the amount of structural (white matter) connections between regions (SC). The relationship between fALFF and measures of connectivity is not well defined; however, the relationship between SC and FC has been explored widely across health and disease/disorder (Kuceyeski et al. 2019; Caeyenberghs et al. 2017, 2013; Cabral et al. 2011; Gu et al. 2021; Castro‐Chavira et al. 2016; Fotiadis et al. 2024; Baum et al. 2020; Liégeois et al. 2020; Zarkali et al. 2021; Cocchi et al. 2014). We found that longitudinal change in fALFF and FC + SC were negatively correlated in the cortex, wherein regions with larger decreases in FC and/or SC over time also tended to have increases in fALFF. We conjecture that increasingly functionally and structurally disconnected regions had increasing (decoupled) neuronal activity, either as an effect of broad axonal degradation or less regulation from outside inputs.

ALFF and FC have been positively correlated in autism (Itahashi et al. 2015) and in controls and individuals with mild cognitive impairment (Weiler et al. 2014; Di et al. 2013). However, in both studies, this was limited to certain regions; most regions did not have such correlations between the fMRI metrics. Prior research has found a positive correlation in controls between SC and relative low frequency power (Fallon et al. 2020), a related measure to fALFF, a correlation we also found to a degree in HC and TBI subjects when we looked at regional values (not the TBI related effects on the metrics, see Figure S2). As for the relationship between SC and FC, a longitudinal study found that at the acute timepoint (within 7 days of injury), FC and SC were negatively related (Dall'Acqua et al. 2017). Similarly, another study at the chronic timepoint showed decreased SC in the cingulum was correlated with increased FC within the frontal node of the DMN (Palacios et al. 2013). Our results are somewhat contradictory to these prior findings, but our analysis is inherently different, as we correlate regional node strength changes in structure and function due to TBI.

## 5 Limitations and Future Work

There are some important limitations to this study, the first and foremost being the small sample size, particularly for PET imaging (only seven TBI subjects had both subacute and chronic PET and MRI data). PET is expensive and difficult to collect and analyze and involves intravenous injection of a radiotracer, so it poses more risk to individuals. (f)MRI and PET are modalities that suffer from low SNR, particularly in the subcortex, making it more difficult to obtain reliable measures in those regions. Secondly, as with many TBI studies, the individuals included in our cohort have wide heterogeneity in injury location, etiology, and severity. Although there is a range of injury severity in our cohort, mild TBI was more common in our study (see Table S1) so any of our findings here may not reflect potentially different mechanisms in severe TBI. Both the small sample size and the heterogeneity of the cohort contribute to limited statistical power; as a result, the effects reported here do not survive correction for multiple comparisons and should be interpreted with caution and repeated in a larger sample. Thirdly, distributions of GABA‐A receptors (which is where FMZ‐PET binds) vary across the brain and thus our measure of BP_*ND*_ may be impacted by this heterogeneity (Rajkumar et al. 2021).

Our main longitudinal analysis also did not have one important factor, namely, an equivalent measure to the chronic time point for the non‐injured controls. Including such information would allow isolation of TBI‐related changes over time compared to what could be test–retest measurement noise or natural variability in individuals, as well as the natural effects of aging in these measures. It would be imperative to replicate these findings in not only a larger cohort but also including a second measure from controls. Although a second session was available for healthy controls, these data were excluded because healthy controls systematically received a lower PET radiotracer dose at the second scan, which affected BP_*ND*_ estimates and introduced a substantial confound. Accordingly, only first‐session data from healthy controls were included in the present analyses. Also, other work including graph theoretical measures of SC and FC and their inter‐relationship could be used to reveal differing topological changes due to TBI (Pandit et al. 2013; Imms et al. 2019). Finally, future work should look at how relationships between metrics, perhaps within specific networks, are related to various measures of outcome.

## 6 Conclusion

Here, we use longitudinal multi‐modal MRI and FMZ‐PET imaging in TBI in an exploratory analysis to examine cross‐modal relationships during recovery after TBI. We found that individually, patterns of FC, fALFF, SC, and BP_*ND*_ generally agreed with previous unimodal studies. Multimodally, however, the relationships between the metrics revealing TBI effects over recovery proved to be dynamic and complex, with an increasing convergence of MRI and PET markers over time. FC and fALFF, as thought to be reflective of possible compensatory mechanisms or changes in neurovascular coupling, are in better alignment with structural and neuronal damage in the chronic stage. Taken together, this longitudinal multi‐modal study is a first step into understanding not only dynamics in recovery, but how multi‐scale structure and function relate across recovery after TBI.

## Funding

This work was funded by NIH grants R01 NS102646 (AK and SS), R01 NS134646‐01A1 (AK), RF1 MH123232 (AK), and R01 AG077576 (SS). This work also used REDCap, which is supported by the Weill Cornell Clinical and Translational Science Center (CTSC) with funding from NIH grant number UL1TR024996.

## Conflicts of Interest

The authors declare no conflicts of interest.

## Supporting information


**Figure S1:** Inter‐modality correlations of the *t*‐values between functional activity strength (fALFF), functional connectivity (FC), and structural connectivity (SC) in all individuals with TBI at the subacute timepoint compared to controls.
**Figure S2:** Spearman correlation between regional SC node strength and fALFF in all available healthy controls and TBI subjects at the subacute timepoint.
**Figure S3:** Brain plots of *η* ^2^ values for TBI group effects from the ANCOVAs.
**Figure S4:** Spearman partial correlations of the whole‐ brain average of each modality's metric (rows) with z‐scored cognitive outcomes (columns).
**Figure S5:** Regional group differences and longitudinal change in functional activity strength (fALFF) when including rapidtide in the preprocessing pipeline.
**Figure S6:** Unimodal and multimodal longitudinal change analysis results in TBI subjects across modalities when including the interval between sessions as a covariate.
**Table S1:** Demographic and clinical data for the TBI group. SDH, subdural hemorrhage; IPH, intraparenchymal hemorrhage; SAH, subarachnoid hemorrhage.
**Table S2:** Demographic data for the HC group.

## Data Availability

The data that support the findings of this study are available on request from the corresponding author. The data are not publicly available due to privacy or ethical restrictions. Code supporting statistical analyses and data visualization are available here (https://github.com/anaradanovic/MultiMod_TBI.git).
