# Supplementary material for: Longitudinal Multimodal Neuroimaging After Traumatic Brain Injury
Source: Hum Brain Mapp. 2026 Apr 27;47(6):e70534. doi: 10.1002/hbm.70534 (PMC13121098; doi:10.1002/hbm.70534)
Supplement: Supplementary file 1 — Figure S1: Inter‐modality correlations of the t‐values between functional activity strength (fALFF), functional connectivity (FC), and structural connectivity (SC) in all individuals with TBI at the subacute timepoint compared to controls. Figure S2: Spearman correlation between regional SC node strength and fALFF in all available healthy controls and TBI subjects at the subacute timepoint. Figure S3: Brain plots of η 2 values for TBI group effects from the ANCOVAs. Figure S4: Spearman partial correlations of the whole‐ brain average of each modality's metric (rows) with z‐scored cognitive outcomes (columns). Figure S5: Regional group differences and longitudinal change in functional activity strength (fALFF) when including rapidtide in the preprocessing pipeline. Figure S6: Unimodal and multimodal longitudinal change analysis results in TBI subjects across modalities when including the interval between sessions as a covariate. Table S1: Demographic and clinical data for the TBI group. SDH, subdural hemorrhage; IPH, intraparenchymal hemorrhage; SAH, subarachnoid hemorrhage. Table S2: Demographic data for the HC group. [file HBM-47-e70534-s001.docx]

**Supplementary information**

**Multimodal correlations large group**

We repeated the multi-modal correlations of TBI-related pathology at the subacute timepoint using the larger dataset of all individuals with subacute fMRI and dMRI data. This was only done with dMRI and fMRI because subjects with both PET and MRI were subjects who also had both sessions of data, therefore, there are no new subjects to analyze in this set. T-values of the coefficient’s effects from the regional subacute unimodal ANCOVA analysis in Figure [2](#_bookmark1) were correlated across the neuroimaging markers via Spearman rank correlation with permutation-based p-values. Correlations were calculated for all 86 cortical, subcortical and cerebellar regions (solid black line) and for 68 cortical regions only (dashed blue line) to isolate correlations in the cortex from whole brain correlations. Results are depicted in Supplementary Fig. [1](#_bookmark4). A similar relationship is observed between FC and SC, such that the TBI-related pathology in FC and SC was positively correlated in the cortex (*r* = 0.32, *p* = 0.01), such that regions with lower FC in TBI compared to HC were also found to have lower SC compared to HC. This was in the same direction as what was found with the smaller cohort in the main text. Both the smaller cohort and the larger cohort had trends for negative correlations between TBI-related fALFF changes and TBI-related SC changes although neither were significant.

**LFO and SC node strength**

Previous work has identified correlations between fMRI-derived low frequency oscillations (LFO) and the structural connectivity strength of the region in non-injured controls.^1,2^ Here, using the non-injured controls (*n* = 14) and TBI subjects (*n* = 40) separately, we correlated each regions’ SC node strength with the same regions’ fALFF values via spearman correlation, and found a significant positive correlation (TBI: *r* = 0.23, *p* = 3.72 * 10^-44^ , HC : *r* = 0.29, *p* = 7.05 * 10^-25^) which agreed with those previous works’ findings (see Supplementary Fig. [2](#_bookmark5)). The p-values reported are permutation based.

**Further ANCOVA results**

Supplementary fig. [3](#_bookmark6) illustrates the *η*^2^ values for the TBI group coefficients in the ANCOVA originally shown as t-stats in Figure [2](#_bookmark1) and Figure [3](#_bookmark2). The top and bottom rows are regional *η*^2^ values for the group term (TBI vs HC) from ANCOVA conducted using all TBI subjects we had available at and, respectively (see Figure [2](#_bookmark1) in main text), while the middle row shows the *η*^2^ values for the TBI group coefficient in the ANCOVA for the subacute time point but for only those with both subacute and chronic data, as in the top row of Figure [3](#_bookmark2).

**Supplementary Figure 1**. **Inter-modality correlations of the t-values between functional activity strength (fALFF), functional connectivity (FC), and structural connectivity (SC) in all individuals with TBI at the subacute timepoint compared to controls.** Subacute time- point inter-modality correlations of the t-values of TBI group effects from ANCOVA analysis via Spearman correlation with permutation-based p-values. Correlation of group effects for whole brain or, where indicated, cortex only (in blue). Cortex-only correlation only shown for comparisons where it differs substantially from the whole-brain correlation. Correlation strength and significance highlighted in bold with a box and asterisks for comparisons with *p<*0.05 (uncorrected). *N* varies by modality, as indicated in each subplot.

**Correlations between global imaging metrics and cognitive measures**

Subjects with TBI and non-injured control participants performed the Attention Network Test (ANT),^3^ a computer-administered measure designed to examine alerting, orienting, and executive attention networks. Mean reaction time and executive attention scores were normalized and corrected for the linear effect of age based on our set of non-injured controls, including those who did not have neuroimaging data (*n* = 67, age 22-86, 41M/26F), and trans- formed to psychometric z-score such that more positive values indicate better performance. Participants also performed a battery of assessments, including: Wechsler Adult Intelligence Scale- Fourth Edition : Coding, Digit Span test, Letter Number Sequencing (LNS), Symbol Search, Trail Making Test A (TMT-A) and B (TMT-B), Stroop test, Rivermead post concussion questionnaire (RPQ), and Glascow Outcome Scale Extended (GOSE). Each cognitive outcome measure (except the GOSE, RPQ, and executive attention) was z- scored using standardized z-scoring procedures. Each imaging metric from each modality was globally averaged across all regions and this global metric was correlated with the various cognitive outcomes via Spearman partial correlation, with sex and age as co-variates. For longitudinal analysis, global average metrics and outcome metrics were computed as a percent change, where the value at chronic was subtracted by the value at the subacute timepoint, divided by the absolute value of the sum of subacute and chronic values. Results are depicted in Supplementary Fig. [4](#_bookmark8). We largely see no significant relationships except for uncorrected significance between BP*_ND_* and executive attention at the chronic timepoint, where individuals with lower BP*_ND_* had better executive attention. The change in BP*_ND_* and fALFF over time was correlated with the change in GOSE over time where more increases in BP*_ND_* and fALFF were related to better GOSE scores. At the chronic timepoint, SC is negatively correlated with TMT-A.


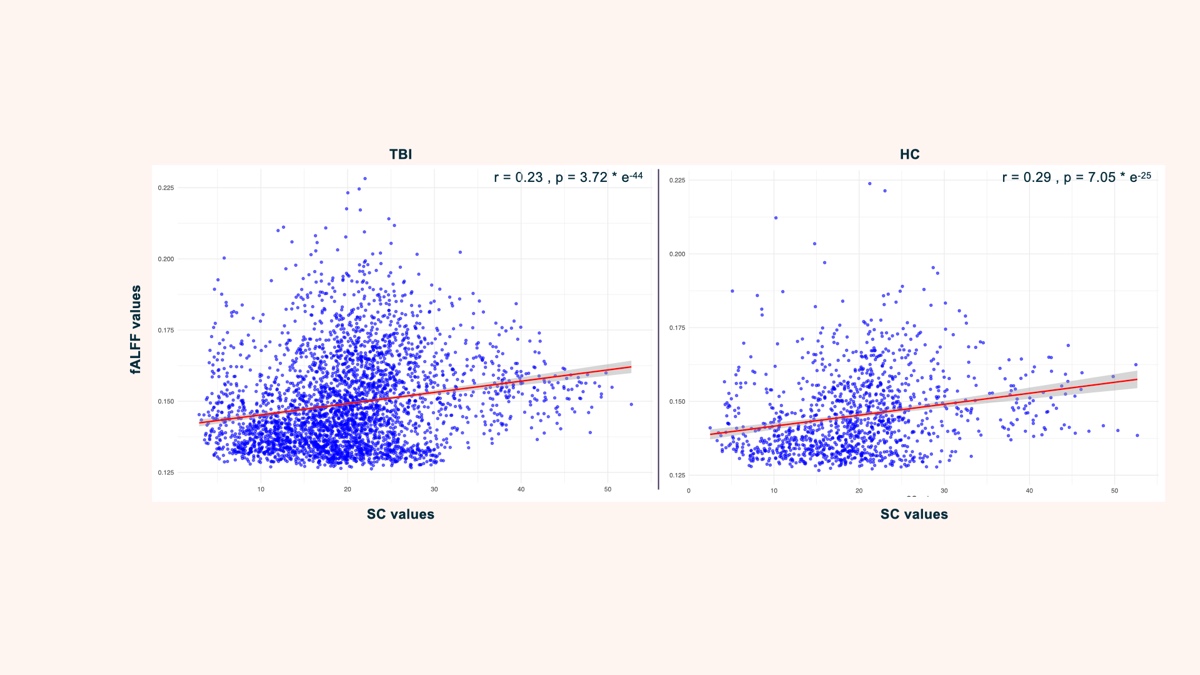


**Supplementary Figure 2**. **Spearman correlation between regional SC node strength and fALFF in all available healthy controls and TBI subjects at the subacute timepoint.** Each point is a region from a specific individual, for all TBI subjects at the subacute time point (left) and healthy controls (right). Spearman correlation was used to examine the strength of the relationship between regional SC node strength and fALFF across all regions and individuals. Correlations are indicated in the upper right hand corner of each figure with associated permutation based p-values.

**Supplementary Figure 3**. **Brain plots of** *η*^2^ **values for TBI group effects from the ANCOVAs.** Top row contains results for the subacute timepoint from the full group of available subjects for the subacute timepoint, while the bottom row contains results for the subjects with chronic data. The middle row is the subacute ANCOVA *η*^2^ for TBI group effects, but for only those subjects with data from both subacute and chronic time points (subset subs). Regions highlighted in green reached significance at (*p<*0.05, uncorrected). *N* varies by modality, as indicated in each subplot.

**TBI**

PET fMRI DTI

| **Session 1** |  | | |
| --- | --- | --- | --- |
| n | 9 | 41 | 40 |
| Age (years) | 42 *±* 14.5 | 50 *±* 17 | 49 *±* 16 |
|  | Range 18-58 | Range 19-86 | Range 19-86 |
| Sex | 7M, 2F | 29M, 12F | 29M, 11F |
| GCS | 10.4 *±* 3.5 | 13.3 *±* 2.8 | 13.3 *±* 2.8 |
|  | Range 8-15 | Range 5-15 | Range 5-15 |
| **Session 2** |  |  |  |
| n | 7 | 16 | 16 |
| Age (years) | 48 *±* 10 | 47 *±* 15 | 47 *±* 15 |
|  | Range 33-58 | Range 19-73 | Range 19-73 |
| Sex | 5M, 2F | 12M, 4F | 12M, 4F |
| GCS | 12 *±* 2.8 | 12.6 *±* 2.8 Range | 12.6 *±* 2.8 Range |
|  | Range 8-15 | Range 7-15 | Range 7-15 |
| **Days between visits** | 296 *±* 64  Range | 253 *±* 69  Range | 253 *±* 69  Range |
|  | 208-409 | 147-409 | 147-409 |
| **CT Initial Findings** | SDH/IPH/SAH  4/1/4  * missing radiological data on 2 subjects | SDH/IPH/SAH  22/9/23 | SDH/IPH/SAH  21/9/23 |

**Supplementary Table 1**. Demographic and clinical data for the TBI group.“SDH” : Subdural hemorrhage, “IPH” : Intraparenchymal hemorrhage, “SAH” : Subarachnoid hemorrhage

**Supplementary Figure 4**. **Spearman partial correlations of the whole- brain average of each modality’s metric (rows) with z-scored cognitive outcomes (columns).** Spearman correlations accounted for age and sex. Global average values for every TBI subject available at both timepoints were included in the top row (Subacute) and bottom row (Chronic). Correlations of change in global metrics vs change in cognitive outcomes over time where computed using percent change for both measures. Correlations reaching uncorrected significance are indicated with an asterisk, none survived corrections.

|  | PET | **HC**  fMRI | DTI |
| --- | --- | --- | --- |
| **Session 1** |  |  |  |
| n | 19 | 14 | 14 |
| Age (years) | 44 *±* 13 Range 22-65 | 56 *±* 17 Range 23-86 | 56 *±* 17 Range 23-86 |
| Sex | 12M, 7F | 9M, 5F | 9M, 5F |

**Supplementary Table 2**. Demographic data for the HC group.

**Measured low-frequency oscillation power after RapidTide**

Low-frequency oscillation power has been shown to artifactually increase with in-scanner relaxation, so we used RapidTide^4^ to regress out relaxation-related effects from the fALFF data. Supplementary Figure 5 compares the main-text fALFF results obtained using ANCOVA and linear mixed-effects (LME) models with results obtained after rapidtide^4^ preprocessing. To quantify consistency across preprocessing approaches, Pearson correlations were computed between regional t-values for the group effect (TBI vs. HC) from the ANCOVA and the session effect from the LME, using 1,000 permutations. fALFF results were highly consistent across approaches (r ≥ 0.92, p = 0.002).

**Supplementary Figure 5**. **Regional group differences and longitudinal change in functional activity strength (fALFF) when including rapidtide in the preprocessing pipeline.** At baseline timepoint for both the full dataset and the subset of subjects with both sessions (Subacute - first row), longitudinal change (second row), and follow-up time- point (Chronic - second row). Subacute and chronic timepoints are visualized via t-values of group coefficients from ANCOVA and longitudinal changes are visualized via coefficients of the session effect of linear mixed effects model analysis. *N* varies by timepoint, as indicated in each subplot. Regions with significant (*p<*0.05) values are outlined in green, uncorrected. Permutation pearson’s r and p-values between the results of the original pipeline and including rapidtide are reported.

**Inclusion of days between sessions in longitudinal change analysis**

Although the interval between scans was highly consistent across subjects, we additionally accounted for the number of days between sessions in the longitudinal change analysis. Linear mixed-effects models were fitted with age, sex, and days between scans as covariates. Results from this analysis are shown in Supplementary Figure 6. Supplementary Figure 6A compares regional session effects exhibiting unimodal longitudinal change from this model with those reported in the main text, while Supplementary Figure 6B presents the corresponding multimodal relationships. Results were highly consistent across the two analysis pipelines.

**Supplementary Figure 6**. **Unimodal and multimodal longitudinal change analysis results in TBI subjects across modalities when including the interval between sessions as a covariate. A.)** Regional session effects from linear mixed effects models when the number of days between sessions is included as a covariate alongside age and sex (top row) versus the analysis from the main text (bottom row). Regions with significant (*p<*0.05) values are outlined in green, uncorrected. **B.)** Session effect coefficients indicating change vs change for each pair of modalities are plotted when including the number of days between sessions (top row) versus the original analysis in the main text (bottom row). Correlations are calculated for all brain regions or, where indicated, cortical regions only (in blue). Cortex-only correlations (dashed blue line) are only shown for comparisons where it differs substantially from the whole-brain correlation (solid black line). Correlation strength and *p*-value indicated in bottom corner, asterisk and bold indicate significance (uncorrected, *p<*0.05). *N* varies by modality, indicated under titles of paired modality columns. Each region is colored by the functional network to which it belongs (see legend).

**References**

1. Fallon J, Ward PGD, Parkes L, et al. Timescales of spontaneous fMRI fluctuations relate to structural connectivity in the brain. *Network Neuroscience*. 2020;4(3):788-806. doi:10.1162/netn_a_00151

2. Lee TW, Xue SW. Linking graph features of anatomical architecture to regional brain activity: A multi-modal MRI study. *Neuroscience Letters*. 2017;651:123-127. doi:10.1016/j.neulet.2017.05.005

3. Fan J, McCandliss BD, Sommer T, Raz A, Posner MI. Testing the efficiency and independence of attentional networks. *Journal of Cognitive Neuroscience*. 2002;14(3):340-347. doi:10.1162/089892902317361886

4. Korponay C, Janes AC, Frederick BB. Brain-wide functional connectivity artifactually inflates throughout functional magnetic resonance imaging scans. *Nat Hum Behav*. 2024;8(8):1568-1580. doi:10.1038/s41562-024-01908-6
